# Influence of reward and location on dogs’ behaviour toward an interactive artificial agent

**DOI:** 10.1038/s41598-023-27930-8

**Published:** 2023-01-19

**Authors:** Svenja Capitain, Ádám Miklósi, Judit Abdai

**Affiliations:** 1 Department of Comparative Zoology, Institute for Evolution and Ecology, University of Tübingen, Tübingen, Germany; 2 ELKH-ELTE Comparative Ethology Research Group, Budapest, Hungary; 3 Department of Ethology, Eötvös Loránd University, Budapest, Hungary

**Keywords:** Learning and memory, Motivation, Reward, Social behaviour, Animal behaviour

## Abstract

Animal–robot interaction studies provide outstanding opportunities to understand the principles of social interactions. Here we investigated whether dogs’ behaviour toward a cooperative artificial agent (Unidentified Moving Object (UMO)) is influenced by receiving a reward directly from the agent, and by variability in the UMO’s location. In a problem-solving task, the UMO either helped dogs to obtain food (Direct Reward Group, DRG) or to fetch an object followed by an indirect reward from the owner/experimenter (Indirect Reward Group, IRG). During the Familiarization, the UMO either started from the same location or changed its starting location in all trials. In the Test phase, dogs faced the same task, but additionally a second, unfamiliar UMO was present. We found that both reward groups gazed at the UMO with decreasing latency during the Familiarization, with the IRG showing more gaze alternations between UMO and hiding location. Dogs showed no preference for either UMO in the Test phase but looked at the familiar UMO sooner if it had changed its location during the Familiarization. Thus, direct reward is not necessary to elicit elements of socially competent behavior in dogs, but variability in its motion may be important to improve the UMO’s animacy and promote flexible learning.

## Introduction

Though the study of social behaviour is an integral part of ethology, it is challenging to develop highly reproducible and controlled experiments. The behaviour of individuals depends on a living interactive partner that is difficult to manipulate and control for a longer period. Due to their variability in experience, personality and underlying biases, the interactive partners are unlikely to behave the same way with a test subject repeatedly which makes designing reliable and replicable experiments difficult^1^. Artificial stimuli have been providing a solution for this issue for decades (e.g.^2^). By resembling the usual interactive partner to a certain degree in embodiment or behaviour, particular behaviours can be evoked in the subject and studied comparably to natural conditions^1^. But even those reach their limits when it comes to complex behaviour patterns, interactive reactions and multiple modalities^3,4^. During the last decade, autonomous, semi-autonomous and remotely controlled robots have become increasingly popular as artificial agents in this undertaking^1,3,5^.

Due to their adaptation to the human social environment and socialisation with humans during development, companion dogs (*Canis familiaris*) are thought to be exceptionally skilful and flexible in heterospecific social interactions^6,7^. With their complex social behaviours, companion dogs are an interesting species to apply the animal-robot interaction framework. In particular, robots not resembling a dog or human provide an exciting possibility for controlled investigations into the characteristics of social interaction that facilitate social behaviour in domestic dogs^8^. To ensure that subjects’ behaviour is not influenced by previous experience with the partner, Gergely and colleagues^8^ suggested using a simple, unfamiliar artificial agent as an interactive partner, now generally referred to as Unidentified Moving Object (UMO). Indeed, several studies seem to suggest that dogs display social behaviour toward socially interactive UMOs. First and foremost, Gergely and colleagues^8^ provided evidence that in problem-solving tasks with an inaccessible object, dogs display similar gaze alternations between an inaccessible object and an interactive UMO, and they gazed at the UMO faster with proceeding trials, just as they do with a human partner (e.g.^9,10^). This gazing behaviour has thus widely been characterized as attention-getting behaviour to initiate interaction and solicit help from an adequate social partner^8,9,11^ (for a review see^12^). In contrast to mechanistically moving UMOs, socially interacting UMOs were also more likely to elicit the A-not-B error in dogs^13^ and led dogs to choose the less preferable food quantity in a two-way choice task^14^.

Despite many studies, we know relatively little about the nature of the dog-UMO relationship. However, if we aim to use this novel and promising method to investigate social behaviour, it is pivotal to understand the underlying factors that influence the dogs’ behaviour and drive the interaction. One problem is that in most studies to date, the UMO interacted with the dog by providing food or a toy as a direct reward for the interaction (UMO in^11,15^; humanoid robots in^16,17^). Thus, the main question is whether dogs solely associate the UMO with the obtained reward, similar to a ‘feeder’, or whether they indeed interact with the UMO based on its social behaviour^14,15^. So far, only a few experiments have investigated these explanations. In Abdai et al.^14^, the reward was partially disassociated from the UMO in a control group by having the owner take the retrieved food from the UMO and handing it to the dog. Still, those dogs showed the same behaviour towards the artificial agent as dogs that received the food directly from the UMO.

Several robot studies have pointed to a set of social stimuli that are needed in the interactive agent to facilitate the seemingly social behaviour in dogs. Gergely et al.^8^ provided evidence that dogs only continue to interact with a UMO that showed goal-directedness and contingent reactivity to the dogs’ initiating gazes. Meanwhile, their attention toward mechanistically moving and unresponsive UMOs and humans decreased significantly within five trials despite all partners directly giving the dog rewards. Likewise, the dog-like robot AIBO that did not show reactive or goal-directed behaviour was not able to elicit social interest in dogs^18^. Furthermore, a study on animacy perception by Abdai et al.^19^ showed that even without any prior, direct interaction with the UMOs, dogs displayed a preference for UMOs demonstrating a chasing interaction over those moving independently from each other.

The above described findings suggest that there is a need for the agent to display a set of sign stimuli (e.g. variable, non-predetermined movement pattern) and interactivity (contingent reactivity) to facilitate persistent social interactions with dogs^8,13,14^. These characteristics were also found to be significant for human children to recognize animate displays^20,21^. We thus argue that not the reward itself but the social cues presented by the interactive partner facilitate the successful interaction seen in earlier studies. Based on these stimuli, it is likely that dogs can quickly generalize the socio-cognitive representation they have established through diverse interactions with humans and act socially towards the novel partner^14^.

However, since most of these dog-robot interaction studies have utilized a direct reward coming from the interactive agent, it remains pivotal to assess the role of reward and motivation in the interactions. Irrespective of reward type, some kind of positive outcome is needed to sustain an interaction. Indeed, associative learning plays a role in such social interactions^22^, particularly to support learning about the details of the social behaviour displayed by the novel partner. Instead, the main question to investigate is whether dogs still interact with the UMO if its behaviour is not directly associated with the reward. More precisely, would there be a difference in the dogs’ behaviour if they interacted with an UMO that provides an indirect reward that is decoupled from the UMO by time and space instead of an UMO that provides a direct reward.

In addition, it is also important to study whether the variability in the UMO’s spatial location influences dogs’ behaviour. Earlier research showed that dogs display strong location bias, that is, visiting the same place following repeated exposures, even when the cues for learning were switched. These studies revealed that location often plays a bigger role than object characteristics^23,24^ or human features^25^ during learning processes. For example, Nitzschner et al.^25^ revealed that dogs were less likely to choose the person that had previously been observed to be generous towards their owner if, after several trials, the person switched its place with the ungenerously acting person. In a reverse learning task, dogs learned to discriminate between cues for the presence of food much faster when the cues were spatial (left or right side) instead of having a certain colour and shape^24^. In addition, Gergely et al.^8^ showed that dogs gaze longer at a UMO moving on varying paths than one that retraces the same path over and over. Thus, learning about and recognizing the interactive agent might be overridden by a location bias even in dog-UMO interactions. Exploring whether this is the case is not just crucial for the design of future studies but might also give further insight into which behavioural patterns are important to facilitate dogs’ recognition of and social interaction with a social partner.

The present study therefore investigated the impact reward association and location have on dogs’ behaviour towards an interactive artificial agent. To facilitate comparisons with earlier studies, we employed a previously utilized experimental set-up wherein the social agent helped the dog to access an inaccessible target object in a problem-solving task. Similarly to those studies, we assessed dogs’ gazing behaviour between the partner and the hiding place (e.g. human as social partner^10^; UMO as social partner^8,13^. The UMO helped dogs to either (1) retrieve a piece of food, or (2) to fetch a bottle at the command of the owner (between-subject design). While in the former group, dogs received a food reward from the UMO directly (direct reward), in the latter case, the reward was decoupled from the UMO in both time and space (indirect reward), that is, instead of the UMO providing a direct food reward to the subject, dogs received only social reward from the owner after completion of the task, with half of the fetching group additionally receiving food from the experimenter well after completing the task. In the Familiarization phase, the UMO either started from the same place in all trials or switched its location between two places from trial to trial. In the subsequent Test phase, dogs faced the same problem-solving task, but now a second, unfamiliar UMO was also present that did not help the dogs to obtain the target (food or bottle). This second UMO was placed in the familiar UMO’s previous starting location, while the familiar UMO was moved to another starting location.

We hypothesised that dogs recognize the agent as an interactive partner based on the social cues it presents. Thus, we expected dogs to display the same type and same amount of communicative gazing signals toward the UMO as reported for problem-solving tasks with adequate social partners (e.g., humans^10^), irrespectively of the nature of the reward (direct/indirect). We also expected dogs to gaze mainly towards the UMO that previously helped them, rather than towards a novel, unfamiliar UMO partner when both are present. However, based on dogs’ tendency to learn faster about the location rather than characteristics of the partner/marker, we also assumed that dogs would gaze at the unfamiliar UMO placed in the familiar UMO’s previous position more often if the familiar UMO did not vary its location during their familiarization.

## Results

### Latency of first gaze at the UMO

#### Familiarization phase

**Figure 1:**
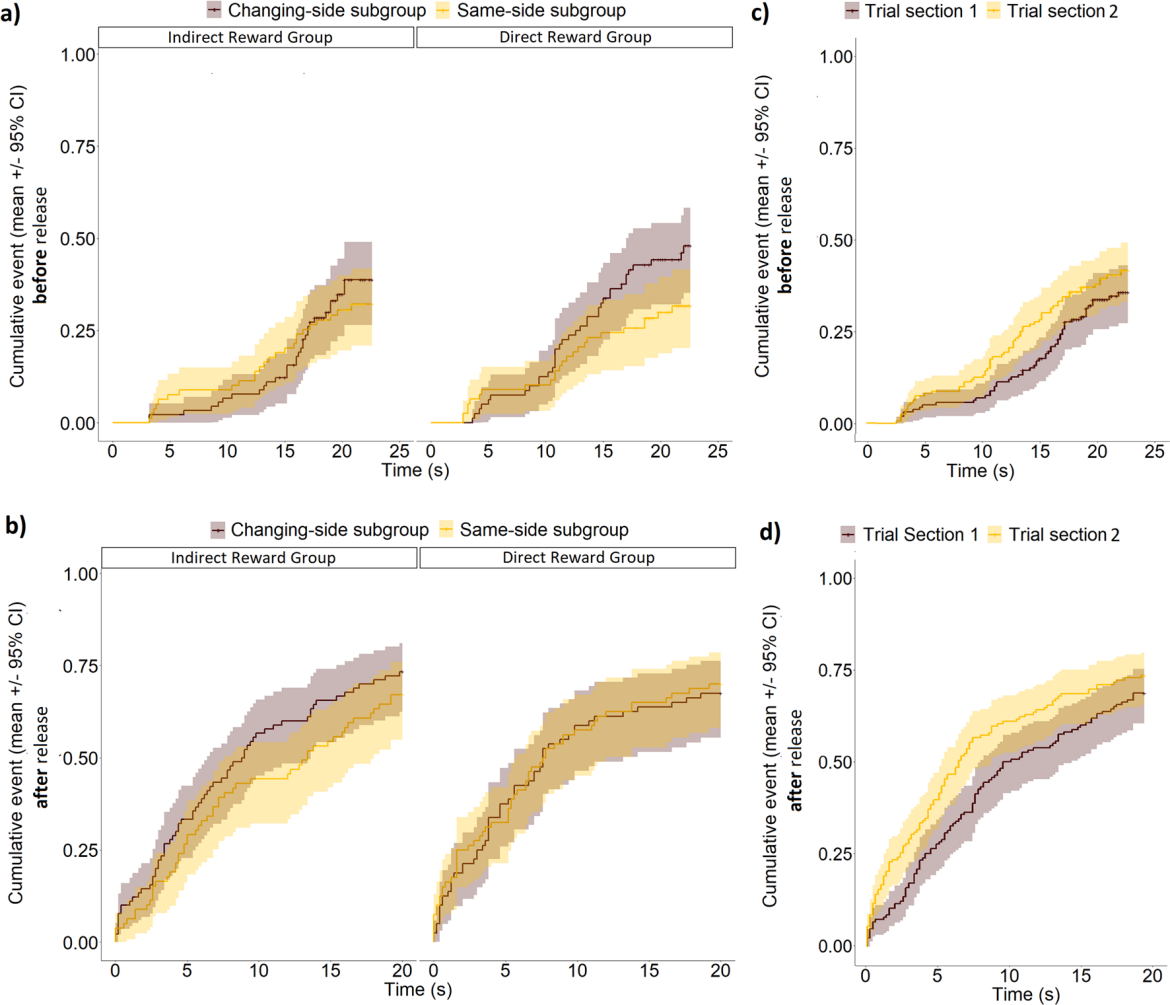
Latency to gaze at the UMO in the Familiarization phase. Reward group and Location subgroup (and their interaction) had no effect on the latency (**a**) before or (**b**) after release. Meanwhile, dogs gazed at the UMO sooner (**c**) before and (**d**) after release in the second (Trial section "Results") compared to the first (Trial section "Introduction") half of the Familiarization phase.

In the Familiarization phase, the latency to gaze at the UMO before or after release was not influenced by the reward group (IRG vs. DRG) and whether the UMO started from the same side or switched sides continuously (mixed effects Cox-regression, LRT: *before release*: Reward group × Location subgroup, $\chi_{1}^{2}$ = 0.51, *P* = 0.477, Reward group, χ^2^(1) = 0.60, *P* = 0.438, Location subgroup, $\chi_{1}^{2}$= 1.23, *P* = 0.268; *after release*: Reward group × Location subgroup, $\chi_{1}^{2}$ = 0.75, *P* = 0.387; Reward group, $\chi_{1}^{2}$ = 0.12, *P* = 0.725; Location subgroup, $\chi_{1}^{2}$ = 0.10, *P* = 0.754) (Fig. 1a,b).

However, the latency to gaze at the UMO was affected by Trial section (*before release*: $\chi_{1}^{2}$ = 3.35, *P* = 0.067; *after release*: $\chi_{1}^{2}$ = 6.18, *P* = 0.013). In the latter half of the Familiarization phase, dogs did not gaze faster at the UMO before release (Familiarization phase first half vs. second half: *β* ± SE = 0.35 ± 0.19, *P* = 0.066), but they gazed at it significantly faster after release (Familiarization phase first half vs. second half: *β* ± SE = − 0.34 ± 0.14, *P* = 0.013) (Fig. 1c,d). The type of UMO used had no effect (Familiar UMO: *before release*, $\chi_{1}^{2}$ < 0.01, *P* = 0.989; *after release*, $\chi_{1}^{2}$ = 1.95, *P* = 0.162).

#### Test phase

**Figure 2:**
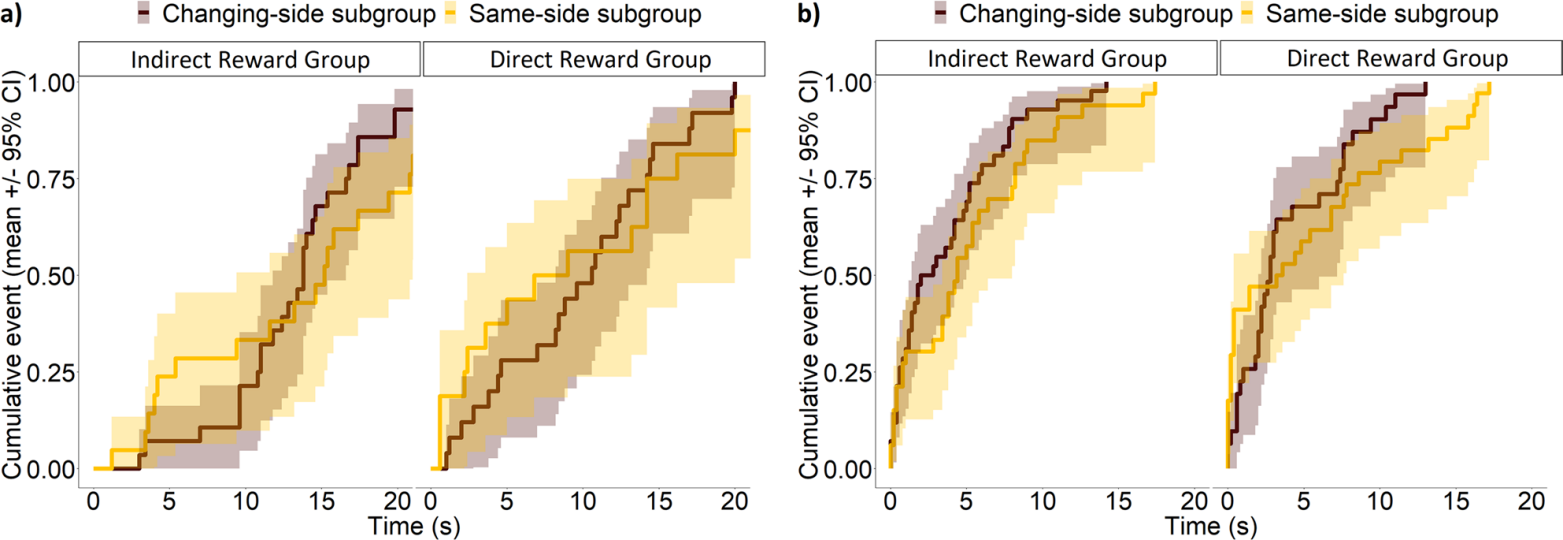
Latency to gaze at the UMO in the Test phase. Reward group and Location subgroup (and their interaction) had no effect on the latency (**a**) before or (**b**) after release.

Similarly, dogs in different reward groups (IRG vs DRG) did not differ in their latency to gaze at the UMO in the Test phase (mixed-effects Cox regression, LRT: *before release*: Reward group × Location subgroup, $\chi_{1}^{2}$ = 0.08, *P* = 0.781; Reward group: $\chi_{1}^{2}$ = 2.40, *P* = 0.121; *after release*: Reward group × Location subgroup, $\chi_{1}^{2}$ = 1.18, *P* = 0.277; Reward group, $\chi_{1}^{2}$ < 0.01, *P* = 0.976) (Fig. 2). Before release, dogs did not gaze at the familiar UMO sooner than at the unfamiliar UMO (First gaze × Reward group × Location subgroup: $\chi_{1}^{2}$ = 0.33, *P* = 0.563, First gaze × Reward group × Trial section: $\chi_{1}^{2}$ = 1.81, *P* = 0.179, First gaze × Location subgroup × Trial section: $\chi_{1}^{2}$ < 0.01, *P* = 0.998). The type of UMO used as the familiar one did not influence the latency to gaze at the UMO in the Test phase (Familiar UMO: *before release:* $\chi_{1}^{2}$ = 0.93, *P* = 0.334, *after release:* $\chi_{1}^{2}$ < 0.01, *P* = 0.989).

**Figure 3:**
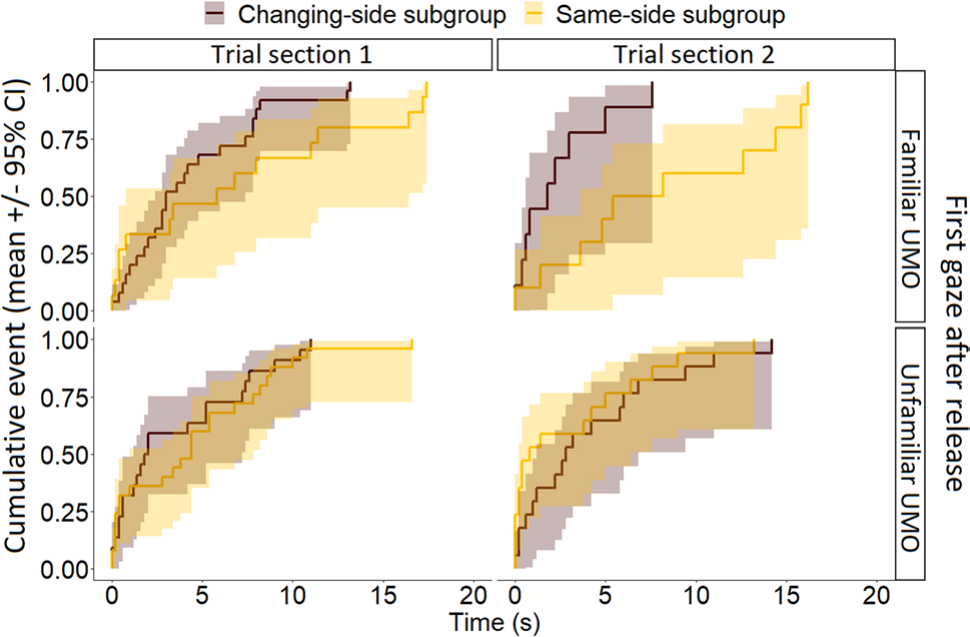
Significant three-way interaction on the latency of first gaze at a UMO after release in the Test phase. Shown are the combined effects of Location subgroup (Changing-/Same-side), First gaze (at familiar/unfamiliar UMO) and Trial section (first/second half of the Test phase).

However, whether the UMO started from the same side or switched sides continuously in the Familiarization phase made a difference in dogs’ gazing behaviour after being released in the Test phase. We found a three-way interaction effect of location subgroup, trial section, and which UMO dogs gazed at first after release (Location subgroup × Trial section × First gaze: ${\chi }_{1}^{2}$ = 4.32, *P* = 0.038). Dogs in the Same-side subgroup gazed significantly sooner at the unfamiliar than at the familiar UMO after release (familiar vs. unfamiliar UMO: *first half: β* ± SE = − 1.08 ± 0.42, *P* = 0.01; *second half: β* ± SE = − 1.99 ± 0.53, *P* < 0.001) (Fig. 3). In the Changing-side subgroup, dogs were significantly faster to gaze at the familiar UMO than dogs in the Same-side subgroup, both in the first three trials (Changing-side vs. Same-side subgroup: *β* ± SE = 1.15 ± 0.49, *P* = 0.02) and the last two trials of the Test phase after release (Changing-side vs. Same-side subgroup: *β* ± SE = 2.25 ± 0.64, *P* < 0.001). Further, dogs in the Changing-side subgroup gazed significantly sooner at the familiar than at the unfamiliar UMO in the latter half of the test phase (familiar vs. unfamiliar UMO: *β* ± SE = 0.98 ± 0.48, *P* = 0.034).

### Analysis of dogs’ first gaze at the UMO(s)

**Figure 4:**
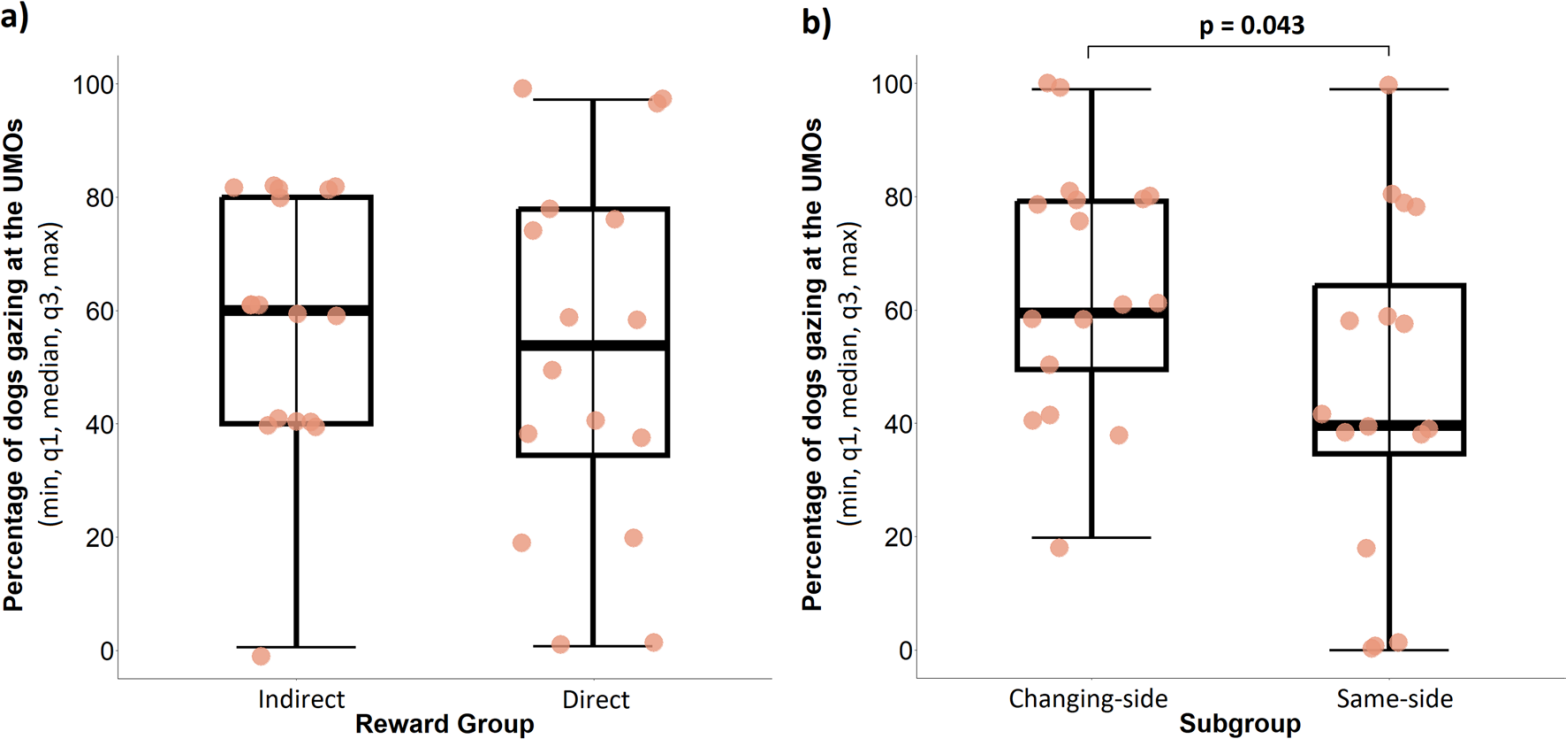
Effect of (**a**) Reward groups and (**b**) Location subgroups on the likelihood of dogs gazing at a UMO before release in the Test phase. The *p*-value indicates the difference between the Location subgroups. For the Reward groups, no post-hoc analysis was conducted because it did not have a significant effect in the model.

The reward group and its interaction with the location subgroup, the fixed effects of trial section and which UMO was the familiar one, did not influence whether dogs gazed at any of the UMOs before release in the Test phase (binomial GLMM, LRT: Reward group × Location subgroup: $\chi_{1}^{2}$ = 1.26, *P* = 0.261; Reward group: $\chi_{1}^{2}$ = 0.21, *P* = 0.644; Trial section, $\chi_{1}^{2}$ = 0.21, *P* = 0.644; Familiar UMO, $\chi_{1}^{2}$ = 1.52, *P* = 0.218). However, location subgroups had a significant effect on this behaviour ($\chi_{1}^{2}$ = 4.01, *P* = 0.044). Dogs in the Changing-side subgroup were significantly more likely to gaze at either of the UMOs before release than dogs in the Same-side subgroup (Changing-side vs. Same-side subgroup: *β* ± SE = 0.83 ± 0.41, *P* = 0.043) (Fig. 4). No such effect was found after the dog was released (Reward group × Location subgroup: $\chi_{1}^{2}$ = 0.75, *P* = 0.385, Reward group: $\chi_{1}^{2}$ = 0.66, *P* = 0.415, Location subgroup: $\chi_{1}^{2}$ = 0.32, *P* = 0.569, Trial section: $\chi_{1}^{2}$ = 0.62, *P* = 0.432, Familiar UMO: $\chi_{1}^{2}$ = 0.77, *P* = 0.381).

**Figure 5:**
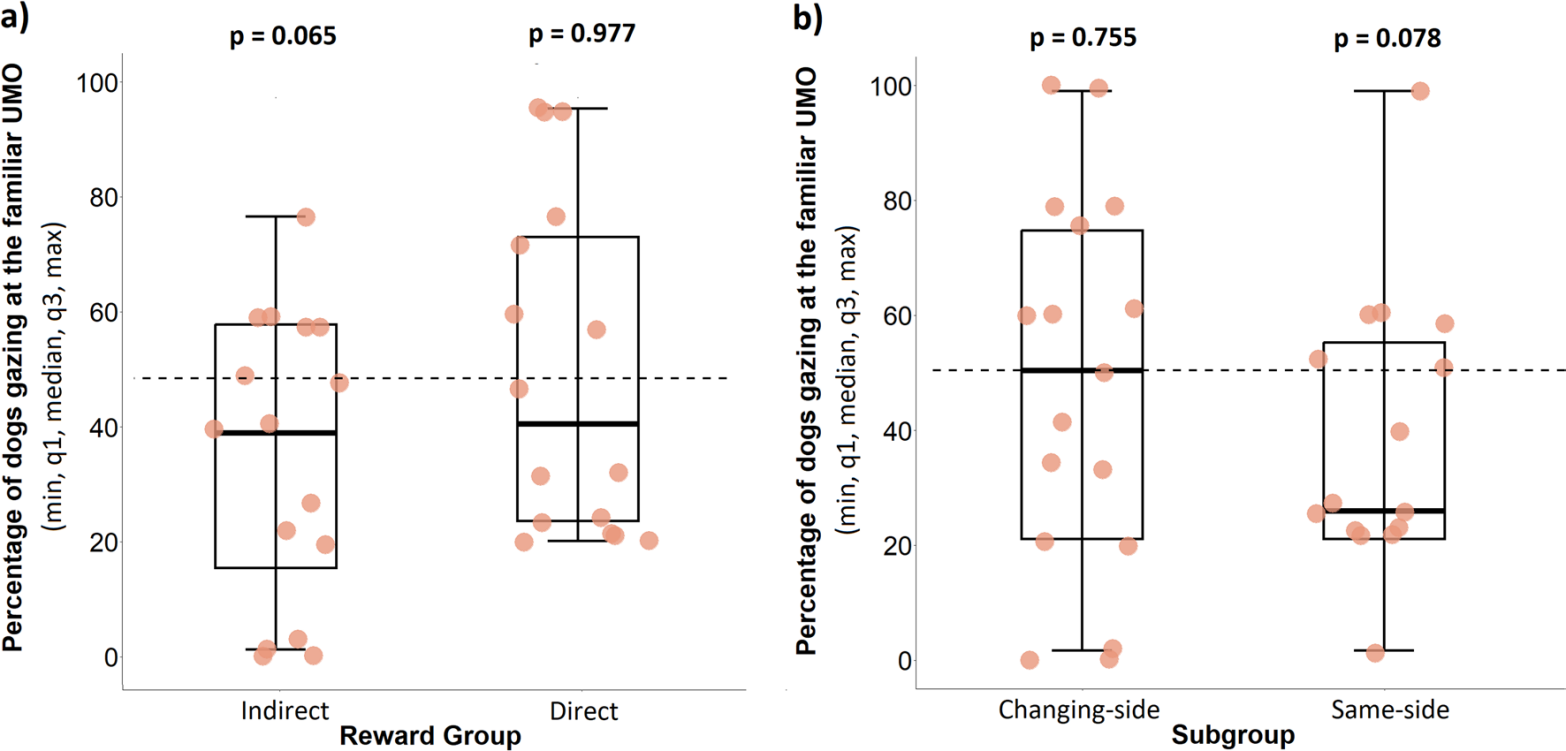
Percentage of dogs gazing first at the familiar UMO in the (**a**) Reward groups and (**b**) Location subgroups after release. The dashed line indicates the chance level of gazing at the familiar UMO first 50% of the time which would be the result if the dogs did not have a preference for either UMO. The *p*-values indicate whether dogs first gazed at the *familiar* UMO significantly above or below chance.

Overall, dogs were not more likely to direct their first gaze at the familiar UMO instead of the unfamiliar one before (Wilcoxon signed rank test: *N* = 33, *z* = − 0.68, *P* = 0.499) or after release (*N* = 33, *z* = − 1.36, *P* = 0.174) in the Test phase. Likewise, there was also no preference towards either of the UMOs in the reward groups and location subgroup before release (DRG: *N* = 16, *z* = − 0.92, *P* = 0.356; IRG: *N* = 17, *z* = − 0.17, *P* = 0.862; Same-side: *N* = 16, *z* = − 0.94, *P* = 0.345; Changing-side: *N* = 17, *z* = − 0.05, *P* = 0.958). We also did not find a preference of gazing at one of the UMOs first after release in either of the reward groups (DRG: *N* = 16, *z* = − 0.03, *P* = 0.977; IRG: *N* = 17, *z* = − 1.84, *P* = 0.065; Fig. 5a) or location subgroups (Same-side subgroup: *N* = 16, *z* = − 1.76, *P* = 0.078; Changing-side subgroup: *N* = 17, *z* = − 0.31, *P* = 0.755; Fig. 5b).

### Gaze alternation between the UMO and the cage

#### Familiarization phase

**Figure 6:**
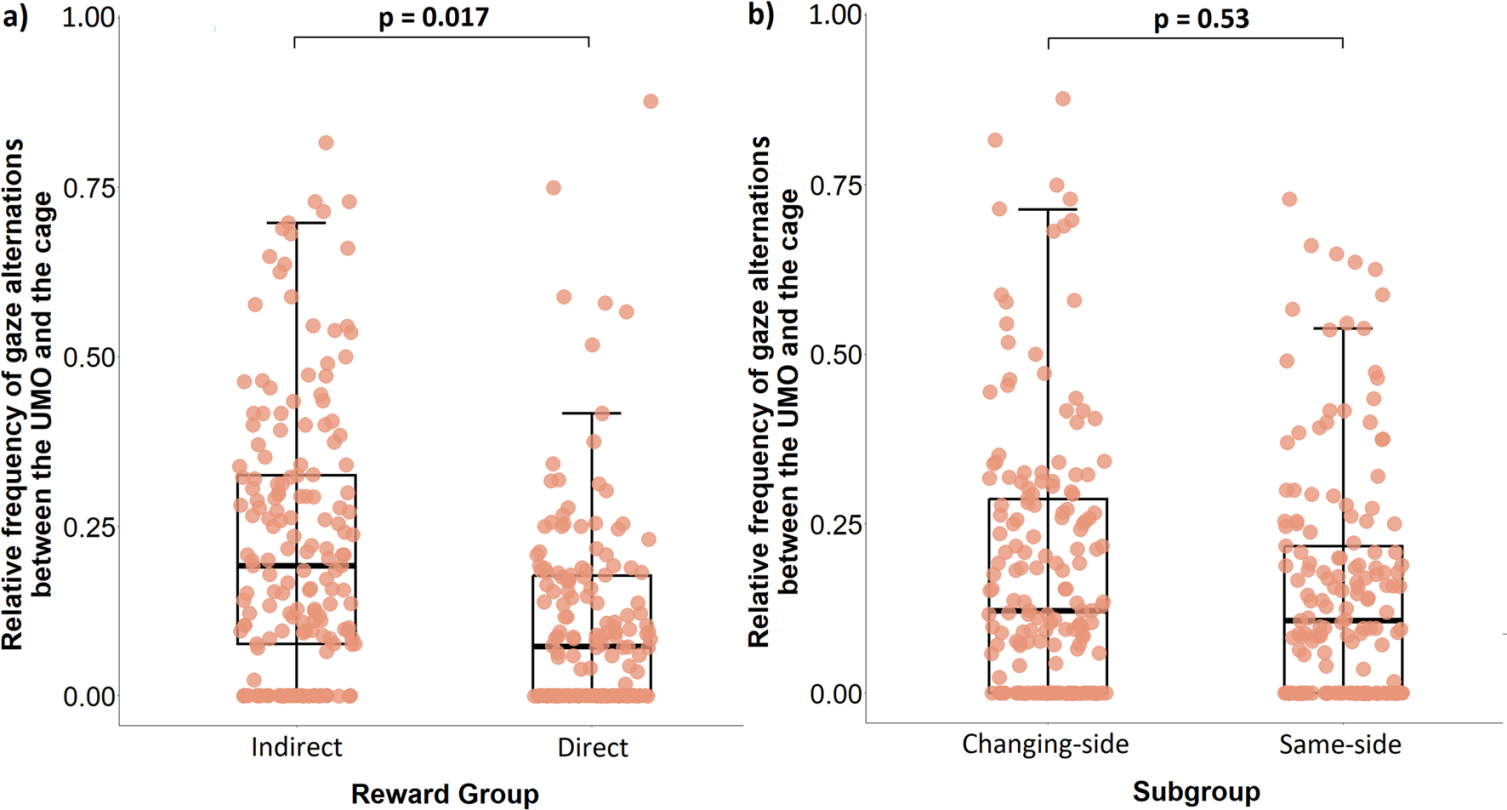
Effects on the relative frequency of gaze alternations between UMO and cage in the Familiarization phase. The p-values indicate the effect between the (**a**) Reward groups and (**b**) Location subgroups on the relative frequency of gaze alternations after release.

In the Familiarization phase, the reward group had an effect on the frequency of gaze alternation (GLMM, LRT: ${\chi }_{1}^{2}$ = 4.91, *P* = 0.003) with dogs in the IRG alternating their gaze between the UMO and the cage significantly more often than subjects in the DRG (IRG vs. DRG: *β* ± SE = 0.10 ± 0.04, *P* = 0.017) (Fig. 6). Whether the UMO started from the same side or switched sides continuously, or which UMO was used as the familiar one did not have an effect (Location subgroup: ${\chi }_{1}^{2}$ = 0.04, *P* = 0.531; Familiar UMO: ${\chi }_{1}^{2}$ = 0.39, *P* = 0.531). In addition, there was a marginal difference in the relative frequency of gaze alternations between the UMO and cage between the first and second part of the Familiarization phase (Trial section: ${\chi }_{1}^{2}$ = 3.45, *P* = 0.063) with a marginal increase of alternations in the second half (Trial section "Introduction" vs. 2: *β* ± SE = 0.02 ± 0.01, *P* = 0.063).

#### Test phase

Regarding the occurrence of gaze alternation in the Test phase, we found an interaction effect of which UMO was moving and whether dogs alternated their gaze between the cage and the familiar or unfamiliar UMO (binomial GLMM, LRT: UMO dogs gazed at × UMO moving, ${\chi }_{1}^{2}$ = 56.15, *P* < 0.001). Dogs were significantly more likely to alternate their gaze between the moving UMO and the cage than the non-moving UMO and the cage (gazing at familiar vs. unfamiliar UMO: when the familiar UMO was moving: *β* ± SE = 1.92 ± 0.49, *P* < 0.001; when the unfamiliar UMO was moving: *β* ± SE = − 2.46 ± 0.4, *P* < 0.001). The other variables did not have an influence on dogs’ gaze alternation (Reward group × Location subgroup: ${\chi }_{1}^{2}$ = 0.01, *P* = 0.923; Reward group: ${\chi }_{1}^{2}$ = 1.44, *P* = 0.230; Location subgroup: ${\chi }_{1}^{2}$ = 0.44, *P* = 0.506; Trial section: ${\chi }_{1}^{2}$ = 0.11, *P* = 0.742; Familiar UMO: ${\chi }_{1}^{2}$ = 0.70, *P* = 0.409).

## Discussion

**Figure 7:**
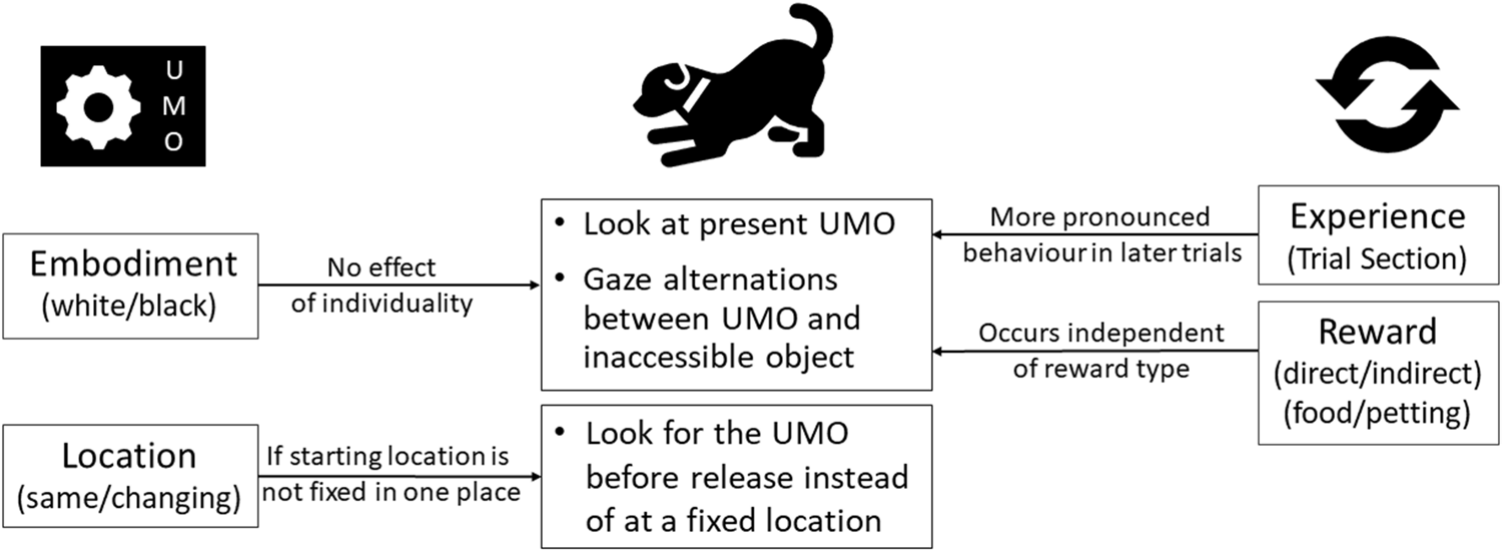
Summary of the investigated factors on the dog’s behaviour. We investigated specific aspects of the UMO (left column) and the procedure (right column) on dogs’ behaviour (middle column).

The goal of this study was to evaluate the impact reward association and location have on dogs’ behaviour towards an interactive artificial agent (for the summary of our main results see Fig. 7). Our results support our main hypothesis that the dogs engage in interaction with the UMO irrespective of whether the positive outcome (reward) was directly associated with the UMO or occurred much later when the immediate interaction with the UMO was over. During the ten Familiarization trials facing the problem-solving task, dogs alternated their gaze with increasing frequency between the UMO and the cage, and gazed at the UMO more rapidly irrespective of reward group. Comparably to the unsolvable tasks where the owner helps the dog to reach the target (e.g.^9,10^), dogs displayed attention-getting behaviour by gazing at the UMO as well as directional actions by gazing at the cage with the desired object (either food or bottle). Gaze alternations are widely considered as communicative attempts by the dog to initiate interaction from an attentive social partner (for review see^12^). Likewise, a study specifically testing different components of gazing behaviour in pet dogs confirmed that gaze at the social partner (in their case: owner), gaze at the desired object (in their case: food), and gaze alternation are socially used by pet dogs and meet the criteria for communication and audience effect^26^. We thus argue that the dogs in the previous dog-UMO studies did not interact with the UMO solely because it provided them with a direct reward, but because it displayed behaviours generally shown by social agents.

Likewise, the decreased latency of gazing at the UMO at the end of the Familiarization phase can be explained by the UMO’s reactivity to dogs’ gaze. Interactivity and contingent reactivity have been identified as crucial behaviour for UMOs to be recognized as an interactive agent^8,13^. In accordance with those studies, we suggest that the UMO’s animate and goal-directed behaviour (as sign stimuli) in combination with the UMO displaying similar interactive behaviour as a human by helping the dog to retrieve the inaccessible object, allowed the dogs to quickly generalize their socio-cognitive representations, and develop and employ expectations about the UMO’s behaviour as an interactive partner. This is especially interesting as the UMO’s embodiment does not seem to be a crucial factor for the dogs’ social behaviour. Not only was there no influence of the different colours and shapes of UMOs in our study and a similar study with four UMOs^27^, but Gergely et al.^8^ also showed that dogs did not discriminate between UMOs with and without eye spots. Behaviour thus seems to be enough for such socio-cognitive representations to occur.

Although no major difference was found between dogs’ latency to gaze at the UMO in the direct and indirect reward groups, we did find a difference in the intensity of the interaction. Dogs that had to fetch the bottle for an indirect reward alternated their gaze more often between the cage and the familiar UMO than dogs trying to obtain a direct food reward, irrespectively of whether the fetching dogs received a food reward from the experimenter or not. A limitation of our study might come into play here since the dogs tested in the two reward groups were different based on their ability to fetch an object *reliably*. This might be based on both the general behaviour of the dog (e.g. only fetches objects that are important to them) but also their training history (the owner did not train them to fetch an object). Trainability has emerged as stable temperament trait that correlates both strong attention to the owner and task as well as willingness and ability to fetch^28^. Likewise, heavily trained dogs (for agility or search and rescue) showed significantly more gaze alternations than untrained pet dogs in an unsolvable task with their owner^29^. Since the reward groups did not differ in their latency to gaze at the UMO, we argue that these differences between the groups do not necessarily reflect a difference in their social perception of the UMO based on the reward type but might simply indicate a difference in their management of the task. While we did make an effort to keep an owner influence low, we cannot exclude the possibility that owners of IRG dogs might have shown subtle differences in their behaviour compared to DRG owners. However, in a study by Merola and colleagues^30^ dogs showed no difference in their gazing behaviour towards an ambiguous stimulus regardless of whether the owner used a happy or fearful voice and facial expression. We thus argue that the situational influence of the owner was likely not the main reason for the difference between the dogs. At the same time, dogs approached an ambiguous stimulus more often when the positive messaging came from the owner rather than a stranger^31^, and we thus chose to have the owner in the room during the test to provide the dog with enough calmness and confidence to engage in interaction with the UMO in general.

Neither reward groups nor location subgroups displayed a gazing preference toward the familiar or unfamiliar UMO in the Test phase which goes against two of our hypotheses that (1) dogs would display their first gaze mainly towards the UMO that previously helped them, and (2) the place from which the UMO started to move would be reinforced in the Same-side subgroup. One explanation might be that dogs lacked the ability to remember the specific characteristics of the UMO regardless of reward group. Since our dogs were allowed to explore both UMO’s at the beginning of the Test phase, these characteristics included not only visual discrimination, but also differences in sound and smell due to the difference in car model and length of use in the testing session. Indeed, a recent study that has been published since our experimental stage revealed that dogs fail to recognize an individual UMO or human after a longer delay, when they only interacted with the partner for a short time^27^. Similar evidence for the short-term memory can be drawn from Carballo et al.’s study^32^ where dogs needed six trials to reliably differentiate between a male and a female prosocial and antisocial human, and even more when both partners were females. These authors argue that more experience with the partner is needed to facilitate individual recognition^27,32^.

It is also possible that the dogs did remember the helpful UMO but that a novelty preference for the unfamiliar UMO might have cancelled out the first gaze at the familiar partner. This would also explain why we did not find a prevalent location-bias in the Same-side subgroup contrary to previous characteristic vs. location learning studies^23,24^. One difference between these studies and ours is that in the former, the two possible choices and locations were already introduced during the familiarization and learning part, whereas our dogs in the Same-side subgroup were only familiarized with one location during the Familiarization. Adding a new location might have skewed these dogs’ first gaze away from the initial reinforced location.

However, the previous reinforcement of location still might play a role in the dogs’ behaviour during the Test phase. Interestingly, only the dogs in the Changing-side subgroup gazed at the helpful UMO significantly faster than the unfamiliar UMO towards the end of the Test phase. This further supports an initial novelty effect, wherein the Changing-side dogs adapted to gaze at the helpful UMO first towards the end of the Test phase after having experienced the unhelpfulness of the new, unfamiliar UMO at the beginning of the Test phase. This flexibility to adjust to the situation was additionally seen in the Changing-side subgroup being more likely than the Same-side subgroup to gaze at any of the UMOs before the owner even released them. Such a learning effect to circumvent the unhelpfulness of the unfamiliar UMO was not found in the Same-side condition, suggesting that the dogs exposed to the fixed location rule later lacked the flexibility to adjust to the situation.

In addition, the UMO continuously changing its starting location could have also contributed to the Changing-side dogs’ more rapid recognition of the helpful UMO. It has been proposed that the type of motion path of an object is important for early animate-inanimate distinction in humans^33^. Csibra^20^ also showed that variability in the motion of an object without organism-shaped body can facilitate the attribution of goal-directedness in 6.5-month-old human infants (one vs two paths used around an obstacle to reach the goal). All UMOs in the present study displayed several motion characteristics that are indicative of animacy (e.g. starting from rest or changing speed without external cause (see e.g.^34,35^); however, change in the trajectory was only present in the Changing-side subgroup. Thus, we also need to consider that this difference in the behaviour of the UMOs in the Same versus Changing-side subgroup might influence dogs’ behaviour and count as an additional sign stimulus for successful dog-UMO interactions. Altogether, we thus recommend using changing movement patterns for UMOs in future dog-UMO studies to circumvent inflexible learning and promote animatic representation of the UMO.

Lastly, we do have to acknowledge here that the study was performed with pet dogs of owners that signed up to the study voluntarily, a procedure that despite its best intentions, does pose a potential bias in the sample. This is further corroborated by the fact that some dogs had to be excluded due to highly negative behaviour towards the UMO or lack of motivation towards the task at hand. While our results do provide essential clues for the formation of sociability in pet dogs, this needs to be taken into consideration before extending the findings to the full population.

Our study suggests that direct food reward and indirect social reward have the same potential to elicit socially competent behaviour in dogs towards an inanimate agent in a problem-solving task. We thus suggest that it was not just the direct reward association but several features of animacy and agency, such as goal-directedness, contingent reactivity, and variability in the UMO’s motion pattern that have significant importance in facilitating the emergence of a social interaction with the UMO^8,13,19^. Though it seems like dogs interacted with the UMO like with a real social partner, further research is needed to investigate whether this holds true across contexts and whether their behaviour is a reliable indicator of their perception of the UMO. We provide additional evidence that changing the location of the UMO is essential in further studies as it impacts dogs’ communicative behaviour and improves their ability to refer to the familiar UMO over an unknown one. Our findings advance our understanding of interactive social behaviour and we argue for the importance of endowing social robots with a set of sign stimuli that act as inducers of social interactions even in the absence of direct rewarding feedback.

## Methods

### Ethical approval

The National Animal Experimentation Ethics Committee provided ethical approval (PE/EA/1550-5/2019). The experiment followed relevant guidelines and regulations and was performed in accordance with the EU Directive 2010/63/EU. Owners provided written consent indicating voluntarily allowing their dogs to participate in the study and that the test videos can be used for illustrative purposes (including online videos).

### Subjects

Forty-one dogs were recruited from the Family Dog Project database of the Department of Ethology, Eötvös Loránd University, Hungary and through social media. Dogs were older than one year and had no previous experience with remotely controlled cars at the department. Dogs were divided into two groups. They either (1) received a reward directly from the UMO (Direct Reward Group, DRG) or (2) had to bring a bottle from the UMO to the owner to receive pet and praise from him/her (Indirect Reward Group, IRG), thereby decoupling the reward from the UMO by time, distance and delivering agent. The IRG was further divided into two groups because several owners indicated that their dog would not fetch an object repeatedly without food reward: (2a) fetch with just petting and praise by the owner; (2b) fetch with petting and praise by the owner, followed by a food reward received from the experimenter (E) later on. Group assignment was initially based on the owners’ report of the dogs’ fetching reliability, and further verified in the pretest (see below).

Dogs in each reward group were randomly assigned to either the Same-side or Changing-side subgroup which differed in the variability of the UMO’s location in the Familiarization phase (see Procedure).

We excluded eight dogs: two due to technical issues, one displayed distress in the room, two displayed aggressive behaviour towards the UMO (e. g. biting) and three lacked motivation. Thus, in the final analyses, we had 33 dogs from different breeds: 16 dogs in DRG (7 females, mean age (year) ± SD = 5.7 ± 2.8), 8 in IRG with praise and food (3 females, mean age (year) ± SD = 4.5 ± 3.4), and 9 dogs in IRG with just praise (6 females, mean age (year) ± SD = 4.8 ± 3.2). Each subject participated in only one reward group and location subgroup (see Supplementary Data S1). Since the preliminary analysis did not yield a difference between IRG with versus without food reward (see Supplementary Methods), they were grouped together (N = 17) and analysed as one IRG (9 females, mean age (year) ± SD = 4.6 ± 3.2).

### Apparatus

**Figure 8:**
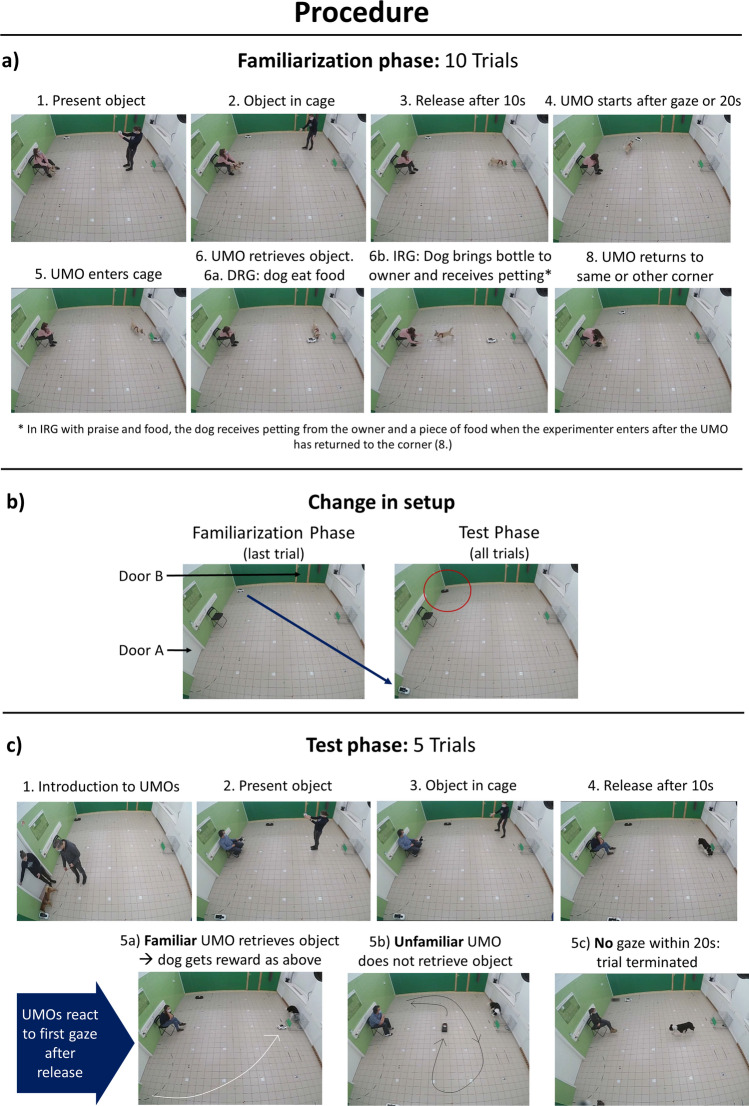
Experimental procedure in the (**a**) Familiarization and (**c**) Test phase, as well as (**b**) the change in setup between the phases. The set up was the same in the Pretest without the cage. The object was a piece of food in the DRG (also the reward) and a plastic bottle in the IRG that they fetched for the owner in return of petting and praise. In the Familiarization phase, the UMO returned to the same starting corner in the Same-side subgroup, while it altered between the two corners in the Changing-side subgroup.

The test was conducted at the Department of Ethology, Eötvös Loránd University, Hungary, in a 6.27 × 5.4 m test room with two doors. In the Familiarization and Test phases, a metal wire-mesh cage (LxWxH: 61 cm × 47 cm × 54 cm) with a front opening of 20 × 13 cm was used. It had a magnet fixed inside to which a plastic dish (13 cm × 9 cm × 5.5 cm) surrounded with metal sheets could be attached, preventing dogs from obtaining the object without the UMO’s help. We used hotdog pieces as food reward, and an empty, flattened, see-through 330 ml plastic bottle with small metal sheets inside to make the bottle heavier, in the IRG. The cage and a chair were placed on opposite sides of the room. The starting position of the UMOs was either in the left or right corner from the chair (Fig. 8).

### Test partners

As UMOs, we used two remote-controlled cars with magnets on their fronts that differed in embodiment and sound. All dogs met one white (36 cm × 18 cm × 13.5 cm) and one black UMO (42 cm × 18 cm × 9.5 or 8.5 cm) (for detailed information see Supplementary Methods) and we counterbalanced between dogs whether the familiar or unfamiliar UMO was black or white. The familiar UMO was introduced to dogs during the Pretest and the Familiarization phases, and retrieved the object from the cage in both the Familiarization and the following Test phase. The second UMO was only introduced in the Test phase (unfamiliar UMO) and moved around the room once before returning to its starting position without retrieving the object (see Procedure). The UMOs were controlled by E from the adjacent room via the live picture of two wide-angle lens cameras (Zoom Q2n).

### Procedure

#### Pretest phase

The UMO was placed in the left or right corner next to the chair (starting side was counterbalanced within location subgroups). First, the dog was allowed to explore the room off-leash while E instructed the owner. The owner then sat on the chair and held the dog in front of them. After E left the room, the UMO moved around the room once, before returning to its starting position. Upon return of E, the dog was released and encouraged to approach the UMO.

Following this, the owner held the dog again. Facing the dog with two meters distance, E showed the plastic bottle or the plastic dish with the food to the dog and called its attention by saying “*(Dog’s name), look!*”. The object was then placed on the floor and E took a step back. The owner released the dog with the command to get the object and the dog ate the food (DRG) or brought the bottle back to the owner (IRG). In the IRG with petting and food, the dog was first petted and praised by the owner like the IRG with just praise group, but was additionally rewarded with a piece of food by E. The owners used the command that the dog was taught to go or search (DRG) or fetch an object (IRG). This procedure was repeated until the dog ate the food or fetched the bottle within 20 s twice in a row. If this was not accomplished within six tries, the experiment was terminated. Dogs that did not fetch as expected but could be motivated by food were switched to DRG (N = 3). Finally, the dog and the owner left the room for a few minutes until the next phase was set up.

#### Familiarization phase

In the Familiarization phase, the familiar UMO helped the dog to retrieve the object from the cage. The starting position of the familiar UMO was the same as in the Pretest. The cage was placed at the wall opposite to the chair (see Fig. 8a). After re-entering through door A, the dog could explore the room until the owner sat down to hold the dog. E showed the object (food or plastic bottle) to the dog, calling their attention with ”*(Dog’s name), look!*”. E attached the object to the magnet inside the cage. After E left the room through door B, the owner waited 10 s before releasing the dog with the command to get the food/bottle. However, the dog was not able to get the object due to the small opening on the cage. The UMO started to move 20 s after the dog was released or earlier if the dog gazed at it. The UMO retrieved the object from the cage by attaching to the dish with the magnets on the UMO’s front. When the UMO left the cage, the owner encouraged the dog to get the object and the dog could eat the food accompanied by verbal praise form the owner (DRG) or take the bottle to its owner and receive petting and praise there (IRG). Meanwhile, the UMO returned to its initial starting position in the Same-side subgroup, or to the opposite corner in the Changing-side subgroup, before E entered again. Only in the IRG with food, the dog received a piece of food from E when she entered. If the dog handled the UMO too roughly (biting, turning over) when it was moving, the owner called the dog back when the UMO started and released the dog again when the UMO came out of the cage (N = 2). This procedure was repeated overall ten times (trials). After the tenth trial, the owner and dog left the room for a few minutes until the room was set up for the Test phase.

#### Test phase

In the Test phase, we placed a novel UMO in the room, thus dogs encountered two UMOs at the same time. The unfamiliar UMO was placed in the corner the familiar UMO had previously started from in the Familiarization phase, while the familiar UMO was moved to the opposite corner (Fig. 8b). This way, we guaranteed that dogs were balanced within location subgroup which UMO they met first. Otherwise the arrangement of the test room remained the same (Fig. 8c). The familiar UMO was not cleaned between the phases to allow for olfactory discrimination on top of the visual differences. First, we introduced the dog to both UMOs and their locations. E and the owner re-entered the room through door A with the dog on a leash. They stopped one meter from the UMO closest to the door. The UMO moved one meter forward, controlled by a second E in the adjacent room, and the dog was encouraged to explore the UMO. The same procedure was repeated with the other UMO in the opposite corner. Afterwards, the dog was free to move for 20 s before sitting back down with the owner. The test procedure was similar to the Familiarization trials: The experimenter showed the object to the dog before placing it in the cage. Then E left the room through door B and the dog was released 10 s later with the command to get the object. Depending on the dog’s behaviour, three events could happen:

1. If the dog first gazed at the familiar UMO, the familiar UMO started to move and retrieved the object from the cage. The dog was rewarded as in the Familiarization phase depending on their reward group, while the familiar UMO returned to the starting position. E re-entered to start the next trial.
2. If the dog first gazed at the unfamiliar UMO, the unfamiliar UMO started to move and circled once around the room before returning to its starting position. The dog did not receive the object, praise or a food reward from the owner or the returning E. After E entered, the next trial started.
3. If the dog did not gaze at either of the UMOs within 20 s after release, the experimenter knocked loudly on the door of the room adjacent to door B and the owner called the dog back. The dog did not receive the object, praise or a food reward. E returned for the next trial.

Overall, we carried out five trials in the Test phase. For a video demonstrating the procedure, see Supplementary Video S1.

### Behavioural variables

All trials were videotaped and the following response variables were analysed for Familiarization and Test phase using Solomon Coder 19.08.02 (by András Péter, https://solomon.andraspeter.com):

#### Latency of first gaze at the UMO (s)

Before release, the latency of dogs orienting their head towards the UMO(s) was measured from showing the object, until the dog was released (maximum time). After release, the latency of dogs orienting their head towards the UMO(s) was measured within the 20 s after being released (maximum time). We indicated with 1 if the gaze occurred within the maximum time, and with 0 if not.

#### Occurrence of first gaze

For both before and after release in the Test phase, we additionally indicated whether the dog gazed first at the familiar or unfamiliar UMO.

#### Gaze alternations between the cage and the UMO(s)

 After release, the number of times the dog oriented their gaze between the cage and a specific UMO and back with a maximum of 0.4 s delay. This was divided by the time between the UMO starting to move and the familiar UMO being 0.5 m from the cage in the Familiarization phase (relative frequency). In the Test phase, gaze alternations were measured as binary variable per trial (alternations occurred in the trial = 1, did not occur = 0) due to the low variability in the frequency of gaze alternations. This was measured between the UMO starting to move and the familiar UMO being 0.5 m from the cage or until the unfamiliar UMO being back at its starting position.

### Statistical analysis

Statistical analyses were carried out using RStudio (version 1.4.1717;^36^) and R software version 3.6.1^37^. For five dogs, fewer trials were recorded due to technical errors or procedural mistakes (for one dog two trials in the Familiarization were not recorded, for one dog only 9 trials were conducted in the Familiarization, three dogs had just 4 trials in the Test). See all data in Supplementary Data S1. For a summary of the behavioural variables and statistical analyses, see Table S2 in Supplementary Methods.

The latency to gaze at the UMO(s) before or after release was fit with mixed-effects Cox regression (package “coxme”) in both phases. To accommodate for slightly different time intervals before release in the Familiarization, the average time owners took to release the dog after the reward was shown (22.6 s) was used as maximum time. For seven out of 327 trials (trials of all dogs), this meant changing an event happened (looking at the UMO before release) to not-happened (not looking) (exact latencies provided in Supplementary Data S1). We used generalized linear mixed models (GLMM) with binomial distribution to explore the influence of our fixed effects (see below) on whether dogs gazed at either UMO in the Test phase before or after release. In the Test phase, we further assessed whether dogs were more likely to direct their first gaze at the familiar UMO rather than at the unfamiliar one. Therein, we used the Wilcoxon signed-rank test with continuity correction (package “stats”) to test whether the percentage of first gazes directed at the familiar UMO was above the chance level of 50%. Trials in which dogs did not gaze at any UMO were excluded from this analysis.

Gaze alternations were analysed as relative frequency in the Familiarization phase. To control for repeated measures and a left-skewed distribution, we used GLMM with gamma distribution (package “lme4”). In the Test phase, we analysed gaze alternations as a binary variable per trial using GLMM with binomial distribution. Trials in which dogs did not gaze at any of the UMOs were excluded from this analysis.

For all mixed models, subjects’ ID was set as a random effect to control for within-subject measurement. The Reward group (DRG/IDR), Location subgroup (Changing/Same-side), embodiment of familiar UMO (black/white), and Trial section (first/second half of the phase) were investigated as fixed effects. For the trial section, the Familiarization was split equally into the first five and second five trials. In the Test phase, the first three trials counted as first half and the last two trials counted as second half. We also included which UMO was moving (UMO moving: familiar/unfamiliar) and which UMO dogs gazed at first (First gaze: familiar/unfamiliar). The model was chosen through stepwise backwards selection using the drop1 function (package “stats”) in the linear models and based on Chi-Square test (ANOVA) in case of the mixed-effects Cox regression. Non-significant variables were excluded from the model, and we report the result of likelihood ratio test (LRT) before exclusion. The “emmeans” package was used for posthoc pairwise analysis in the final models (Tukey adjustment).

Inter-coder reliabilities were investigated on random subsamples of the recordings by a second coder who coded 20% of the subjects. Results indicated acceptable inter-coder reliability for all variables (see Supplementary Methods).

## Supplementary Information

Supplementary Information 1.

Supplementary Information 2.

Supplementary Video 1.

## Data Availability

All data are available as supplementary information.
