# Supplementary material for: Influence of reward and location on dogs’ behaviour toward an interactive artificial agent
Source: Sci Rep. 2023 Jan 19;13:1093. doi: 10.1038/s41598-023-27930-8 (PMC9852237; doi:10.1038/s41598-023-27930-8)
Supplement: Supplementary file 2 — Supplementary Information 2. [file 41598_2023_27930_MOESM2_ESM.pdf]

Electronic supplementary material for:

Influence of reward and location on dogs' behaviour toward an interactive artificial agent

by Svenja Capitain, Ádám Miklósi, Judit Abdai

in *Scientific Reports*

1. Additional information for the methods

1.1 Comparison of the data of dogs in the IRG with and without food reward

Statistical analyses were carried out for all behaviours to test whether dogs within the Indirect Reward Group (IRG) displayed different behaviours depending on whether they received food reward from the returning experimenter after fetching the object, or not (IRG with praise and food vs IRG with just praise) (Table S1). For the behavioural variables and description of the statistical analyses, see the main text.

Table S1. Results of the comparison of dogs in the IRG with praise and food vs IRG with just praise.

Variable	Phase	Time	χ^2	df	*p* value
Relative frequency of gaze alternation	Familiarization	After release	4.94	2	0.08
			IRG with praise and food vs IRG with just praise: β = -0.01, S.E. = 0.06, p = 0.98		
Occurrence of gaze alternations (binary)	Test	After release	1.63	2	0.44
Dogs' first gaze at the UMO (binary)	Test	Before release	0.29	2	0.86
	Test	After release	1.06	2	0.59
Latency of first gaze at the UMO	Familiarization	Before release	0.007	1	0.93
	Familiarization	After release	0.45	1	0.93
	Test	Before release	0.13	1	0.72
	Test	After release	0.90	1	0.34

1.2. Additional information about the test partners

The white UMO was a #32710 RTR Switch Abarth 500 (36 cm x 18 cm x 13.5 cm) with a white plastic cover. The other UMO had a black cardboard cover covered with self-adhesive wallpaper (Figure S1a). Due to technical issues, we used two different cars as basis for the black UMO (only one black car was introduced to each dog): as unfamiliar black UMO, we used a #7304 Traxxas 1/16 Ford Mustang Boss 302 (42 cm x 18 cm x 9.5 cm), but used a #120090 E10 Michele Abbate GrrRacing Touring Car (42 cm x 18 cm x 8.5 cm) as black familiar UMO. The embodiments of these two differed a bit in size but were otherwise identical (see Figure S1b).

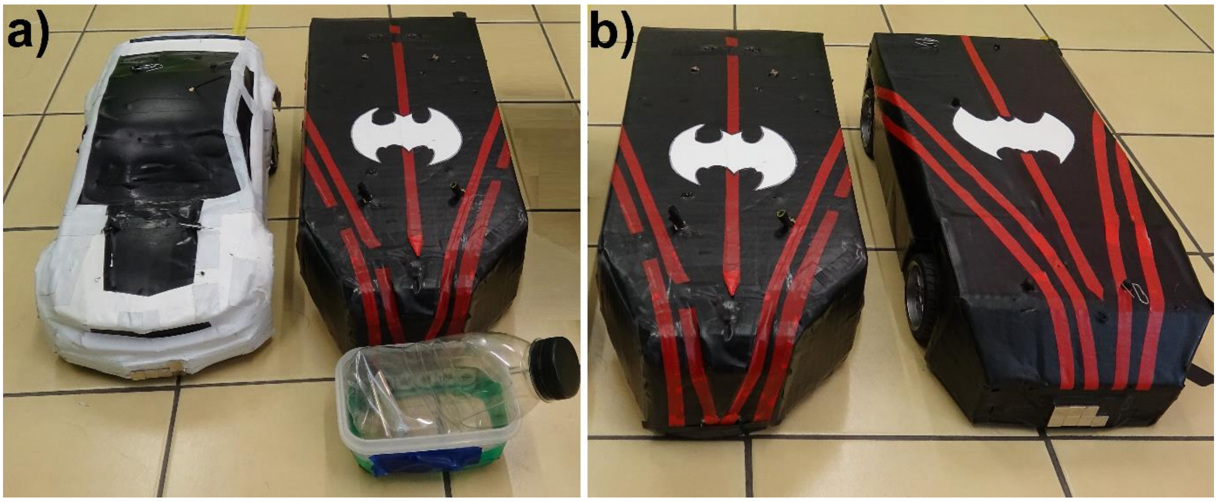

Figure S1. a) The remote controlled cars that were used as familiar and unfamiliar UMOs. The plastic dish could be attached to the UMOs' front and the inside of the cage, and carried the food or the plastic bottle. b) The two versions of the black UMO. The one on the left only acted as unfamiliar UMO in the Test phase, whereas the right one was used in both phases.

1.3. Behavioural variables

Table S2. Behavioural variables and statistical analysis. For all variables, a 95% confidence interval was used. GLMM = generalized linear mixed model

<table>
<tr><th>Variable</th><th>Phase</th><th>Subphase</th><th>Measure</th><th>Test</th><th>Stepwise backwards model selection</th><th>Post hoc</th></tr>
<tr><td rowspan="6">First gaze at the UMO</td><td rowspan="2">Familiarization phase</td><td rowspan="2">Separately for Before release AND after release*</td><td>Latency (s) to first gaze at the UMO</td><td rowspan="2">Mixed-effects Cox regression</td><td rowspan="2">Chi-Square test (ANOVA)</td><td rowspan="2">Tukey adjustment</td></tr>
<tr><td>Binary (gazed at the UMO or not)</td></tr>
<tr><td rowspan="4">Test phase</td><td rowspan="4">Separately for Before release AND After release*</td><td>Latency (s) to first gaze at the UMO</td><td rowspan="2">Mixed-effects Cox regression</td><td rowspan="2">Chi-Square test (ANOVA)</td><td rowspan="2">Tukey adjustment</td></tr>
<tr><td rowspan="2">Binary (gazed at a UMO or not)</td></tr>
<tr><td>GLMM with binomial distribution</td><td>Chi-Square test (ANOVA)</td><td>Tukey adjustment</td></tr>
<tr><td>Binary (gazed at which UMO)</td><td>Wilcoxon signed-rank test with continuity correction</td><td></td><td></td></tr>
<tr><td rowspan="2">Gaze Alternations</td><td>Familiarization phase</td><td>After release**</td><td>Relative frequency</td><td>GLMM with gamma distribution</td><td>Chi-Square test (ANOVA)</td><td>Tukey adjustment</td></tr>
<tr><td>Test phase</td><td>After release**</td><td>Binary (gaze alternations occurred or not)</td><td>GLMM with binomial distribution</td><td>Chi-Square test (ANOVA)</td><td>Tukey adjustment</td></tr>
<tr><td colspan="7">*Before release: measured between showing the object and until the dog was released (maximum time). After release: measured in the first twenty seconds after the dog was released.</td></tr>
<tr><td colspan="7">**After release: measured between the UMO starting to move and the familiar UMO getting within 0.5m of the cage/ the unfamiliar UMO stopping back at its place/the knock on the door.</td></tr>
<tr><td colspan="7"><u>For all mixed models</u>: random effect: Subject's ID; fixed effects: Reward group (DRG/IDR), Location subgroup (Changing/Same-side), embodiment of familiar UMO (black/white), and Trial section (first/second half of the phase). When applicable, which UMO was moving (UMO moving: familiar/unfamiliar) and which UMO dogs gazed at first (First gaze: familiar/unfamiliar). Two- and three-way interactions were also involved in the models</td></tr>
</table>

1.4. Inter-coder reliability

Inter-coder reliabilities were investigated on random subsamples of the recordings by a second coder who coded 20% of the subjects. Regarding the latency to gaze at the UMO, the correlations between the two coders were acceptable for the data of the Familiarization phase before release (r_S = 0.94, $P < 0.001$) and after release (r_S = 0.86, $P < 0.001$), as well as for the Test phase before release (r_S = 0.90, $P < 0.001$) and after release (r_S = 0.73, $P < 0.001$) (Spearman correlation). Inter-coder reliabilities were also acceptable for the frequency of gaze alternation in the Familiarization phase (r_S = 0.81, $P < 0.001$), and for alternations between the familiar UMO and cage (r_S = 0.84, $P < 0.001$), and the unfamiliar UMO and the cage (r_S = 0.91, $P < 0.001$) in the Test phase (Spearman correlation). Regarding the binary data, i.e. which UMO dogs gazed at first in the Test phase, Cohen's kappa indicated acceptable reliability (before release, $\kappa = 0.96$; after release, $\kappa = 0.75$).
